# Assessment of an Aujeszky's Disease Control Strategy in a Highly Prevalent Pig Farm Based on Systematic Vaccination With an Inactivated gE-Negative Marker Vaccine

**DOI:** 10.3389/fvets.2022.852650

**Published:** 2022-04-22

**Authors:** María N. Aznar, Fernando A. Bessone, Rodrigo Segurado, Sergio J. Duffy

**Affiliations:** ^1^Instituto de Patobiología, Centro de Investigaciones Veterinarias y Agronómicas (CICVyA), Instituto Nacional de Tecnología Agropecuaria (INTA), Hurlingham, Argentina; ^2^Sanidad Animal, INTA, Estación Experimental (EEA) Marcos Juárez, Córdoba, Argentina; ^3^Private Veterinary, General López, Santa Fe, Argentina; ^4^Private Consultant, Buenos Aires, Argentina

**Keywords:** Aujeszky's disease, vaccination, inactivated gE-negative marker Bartha K61 vaccine, control strategy, Argentina

## Abstract

Aujeszky's disease (AD) is endemic in Argentina. In 2016, an inactivated gE- negative marker Bartha K61 vaccine (AUSKIPRA^®^ BK) was launched for use, making Argentina the only country to carry out a control strategy plan with it. In the present article, we describe the results of a control program in a farrow-to-finishing farm with high initial AD prevalence (33% in sows), based on the systematic vaccination, detection, and elimination of seropositive pigs, the replacement of sows with vaccinated gilts, and the instauration of artificial insemination. The program was suitable for diminishing the incidence and the prevalence at levels consistent with virus eradication. This situation has been sustained over time. This is the first report of AUSKIPRA^®^ BK efficacy under field conditions.

## Introduction

Aujeszky's disease (AD), also known as Pseudorabies, is a highly contagious disease caused by *Suid herpesvirus* 1, which affects pigs and a wide range of animals. It is economically important and produces severe neurological symptoms and death in piglets, respiratory disease and growth retardation in finishing pigs, and abortion in sows (1). The AD virus is carried by live animals and is generally spread by direct nose-to-nose contact between pigs, and can also be spread through fomites, semen, and the aerosol route. Wind-borne infection from up to 40 km away can occur in areas dense with pigs and farms (2).

Although AD can be found throughout the world, especially in regions with dense swine populations including South America, Asia, and Europe, some countries are free of AD, either because it was never reported (Canada, Australia, and Ireland) or because it was eradicated from their domestic swine populations in the late nineties (Germany, Austria, France, Sweden, Denmark, the United Kingdom, Canada, New Zealand, and the United States).

Biosecurity encompasses the full range of measures aimed to reduce the probability of the introduction (external biosecurity) and further spread of pathogens within the farm (internal biosecurity) and is key to avoid transmission, either between farms or within the farm (3). As there are so many ways in which AD can spread, biosecurity measures to control it have to be broad-based and thorough (4).

Vaccination of susceptible animals is an effective control and eradication strategy. Nevertheless, vaccination can complicate serological surveillance activities if the antibody response induced by vaccination is indistinguishable from that which follows natural infection. This disadvantage can be overcome by the use of DIVA (an acronym for differentiating infected from vaccinated animals) or marker vaccines. The main advantage of DIVA vaccines and their companion tests is the possibility to distinguish between naturally infected and vaccinated animals. The DIVA approach has been applied successfully to AD, avian influenza, and foot-and-mouth disease eradication (5). Regarding AD, intensive vaccination with a marker vaccine glycoprotein E (gE)-deleted has resulted in a decrease of the field virus prevalence in several countries to a sufficiently low level so that culling becomes economically feasible (6). The evolution in the reduction of the circulating field virus can be followed by serological monitoring for antibodies against gE, using commercially available enzyme-linked immunosorbent assays (ELISA) (1, 6).

Modified live vaccines (MLV) are generally more efficacious than inactivated vaccines (IV), particularly when they contain a high virus titer and are adjuvanted (1–3, 5–7).

In Argentina, AD is endemic and was first described in 1979 (8). The National Control and Eradication Plan was established by the National Service for Agrifood Health and Quality (Senasa) (9). This plan comprises two different stages: the estimation of the national AD prevalence and the implementation of the control. Regarding the former, a two-stage random sampling was carried out in 2010. The percentage of infected farms was 19.1% (16.7–21.5%), and the percentage of infected sows was 8.9% (7.5–10.4%). In both cases, the fewer the sows per farm, the higher the prevalence. Regarding the second stage of the National Plan, farms with more than 100 sows must be free from AD (9). Ultimately, the requirement will also apply to farms with fewer sows. Unfortunately, the progress of the plan has not been as quick and thorough as expected. The idea is to progressively reduce the AD prevalence in domestic pigs to levels compatible with eradication but it is unlikely that AD will be eradicated from Argentina in the near future. Regarding the disease in wild pigs, it has also been reported to be present. Abate et al. (10) and Carpinetti et al. (11) found 11 out of 12 (91.0%) and 65 out of 104 (62.5%) reactive animals in Northern Patagonia and the Bahía Samborombón Natural Reserve, respectively. Because of this, all contact between wild and domestic pigs should be avoided to prevent sporadic introductions into the farms.

In the year 2016, Argentina started to use an inactivated gE-negative marker Bartha K61 vaccine (AUSKIPRA® BK (HIPRA S.A). As there are no national AD vaccines, Senasa preferred to import an IV, to minimize the risks involved in the use of MLV concerning adventitious viruses. Regarding the inactivated gE-negative marker vaccine, it is commonly used in combination with an MLV (AUSKIPRA^®^ GN) (12–14). Nowadays, Argentina is the only country that carries out a control strategy plan using only the inactivated AUSKIPRA^®^ BK (HIPRA, personal communication) as vaccination tool. Therefore, there are no reports of its efficacy under field conditions using this product alone, and to the best of our knowledge, there is no experience on the control or eradication of AD using exclusively inactivated vaccines.

Regarding the Argentine pig production, there are approximately 5,377,071 head maintained within 107,221 farms (15). The majority of farms are, principally, in the central and northeast parts of the country. Even in the most productive regions, the pig density is low: the Department of Caseros, in the province of Santa Fe, which has the highest stock of head (101,937 pigs and 13,000 sows), has a density equal to 29.14 head/km^2^ (15). This is a relatively low density compared to other very productive regions of the world such as the southeast of the Netherlands (1,524 head/km^2^), continental Denmark (542 head /km^2^), Catalonia, and Murcia in Spain (734 and 460 head /km^2^) and Brittany, France (428 head /km^2^) (16).

The objective of the study was to describe the results of a control program in a farrow-to-finishing farm with a high initial AD prevalence based on systematic vaccination with an inactivated vaccine, the detection and elimination of seropositive pigs, the replacement of sows with vaccinated gE-negative gilts, and the instauration of artificial insemination.

## Materials and Methods

The study started in 2017 and was performed in a commercial farrow-to-finish farm with 278 sows (Yorkshire-Landrace cross of 1–3 years old and a mean of 2.01 deliveries/year) located in the Department of General López in the province of Santa Fe, Argentina. Although it is a district with one of the highest pig densities, this farm is 2.5 km away from the nearest operation. All the sectors, but gestation, were in confinement. Natural breeding was carried out. The level of the biosecurity of the farm was not very good. The farmer was informed about the purpose of the study and knew that the assessed control strategy would be published in a journal. The farmer provided verbal informed consent for animal blood sampling and vaccination.

As an initial screening, a progressive sampling was performed over 3 months. All sows (n=278), boars (n=4), and 16 gilts were checked with the IDEXX PRV/ADV gE Ab test. This differential test specifically detects the presence of antibodies to the gE antigen in swine serum, which appears with field strains infection, ignoring antibody titters in animals vaccinated with gE-deleted vaccines. AUSKIPRA^®^ BK, an inactivated gE-negative marker Bartha K61 strain vaccine against AD, was used. The implemented control strategy was based on:

- vaccination of sows (two times, every 21 days, and before deliveries) and piglets (at weaning and 21 days after);

- purchase of ELISA gE-negative gilts from Officially AD-free farms vaccinated two times, at 120 and 141 days old, entering the farm 14 days after the second vaccination;

- culling of gE-positive females: a) immediately, if non-pregnant, and b) at the end of their milking period if pregnant;

- culling of all boars and instauration of artificial insemination;

- serological tests monitoring all ELISA gE-negative sows and gilts and 30 finishing pigs every 6 to 9 months.

Also, some basic biosecurity improvements that were feasible for the farmer were implemented, such as the presence of foot baths with disinfectants at the entrance of the different barns, limiting the number of vehicles entering the facility, the control of the pigs' entry, and the control of visitors.

**Figure 1:**
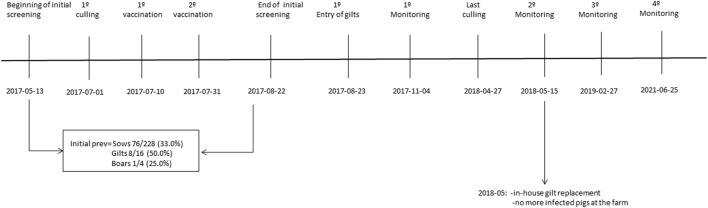
The framework of the control strategies and dates of implementation.

The framework of the actions implemented as control strategies is shown in Figure 1.

## Results

The AD prevalence found at the initial screening was 33% in sows, 25% in boars, and 50% in gilts. The number of serological tests monitoring, their dates, the number of sows and gilts positive and negative to ADV, the total tested and the percentage of positive, and the viral circulation in finishing pigs (VCFP) are presented in Table 1.

**Table 1:** Number of serological tests monitoring, dates, number of sows and gilts positive and negative to ADV, totally tested and percentage of positive pigs, and viral circulation IN finishing pigs (VCFP).

		**Sows**				**Gilts**				**VCFP**
**Monitoring**	**Date**	**+**	**–**	**n**	**%**	**+**	**–**	**n**	**%**	
1	2017-11-04	38	71	109	34.9	4^*^	72	76	5.3	Yes
2	2018-05-15	0	182	182	0.0	0	80	80	0.0	No
3	2019-02-27	0	176	176	0.0	0	75	75	0.0	No
4	2021-06-25	0	186	186	0.0	0	14	14	0.0	No

* *3 gilts entered the farm on 10 August 2017 and 1 on 27 September 2017*.

As seen at the first monitoring, 38 sows out of 109 (34.9%) and 4 gilts out of 76 (5.3%) were positive to the ELISA gE-test. The positive sows had been vaccinated two times. Regarding the seropositive gilts, 3 of them had entered the farm on 10 August 2017 and the other on 27 September 2017 (days of contact with infected sows: 86 and 38, respectively). Also, viral circulation was found in the finishing pigs.

The second monitoring, carried out on 15 May 2018, showed no positive sows or gilts or viral circulation in the finishing sector. The last seropositive animals detected at the initial screening left the farm in January 2018. By May of that year, the farm stopped the external sourcing of gilts by using exclusively in-house gilt replacement. Moreover, the culling finished (no more seropositive animals remained at the facilities).

No positive sows, gilts, or finishing pigs were detected at the monitoring carried out on 27 February 2019 and 5 June 2021.

## Discussion

In the past, most of the countries that were able to eradicate AD used MLV (17, 18) with the exception of France which used both IV in sows and MLV in finishing pigs (19). Nowadays, China uses AUSKIPRA^®^ GN in all pig categories with a booster in sows with AUSKIPRA^®^ BK (12–14). Regarding these two vaccines, both of them are Bartha K61 strains. This strain has a deletion in a region of the viral genome where gE is coded (US region), which prevents it from expressing the gE glycoprotein. Therefore, this strain can be used to obtain the gE-negative marker vaccines. The difference between them is that AUSKIPRA^®^ GN is an MLV, while AUSKIPRA^®^ BK is an IV (chemically inactivated with binary ethylenimine -BEI).”

Four months after the beginning of the control strategy, vaccinating two times (and three times, in some cases), was not enough to avoid transmission among breeding females. Also, there were some gilts that showed seroconversion when they entered the farm, even with three doses, although the majority remained negative. The viral circulation continued at the finishing sector, similarly as described by Liu et al. (12), indicating that the pigs became infected as piglets.

Ten months after the control strategy implementation, the situation was more favorable with no new infections in sows or finishing. Differently from what happened in the Chinese farm described by Liu et al. (12), only one gilt was positive. This may be due to the fact that in our study the replacement was carried out by introducing ELISA negative gilts.

Then, 20 and 48 months after the beginning of the interventions, no seropositive animal was detected.

The strategy implemented has proven to be effective, and control was soon achieved, in part because the positive pigs were removed from the farm as soon as possible, unlike the study by Liu et al. (12). This quick removal of sources of infection was a key aspect in the decline in prevalence. Also, the improvements made in biosecurity, although not exhaustive, helped to reach control and eradication. The fact that not the best biosecurity measures could be taken allows us to emphasize more the effectiveness reached by the enforced vaccination plan.

MLV is generally acknowledged to be more efficacious than IV (1–3, 5–7). However, the latter has shown to be efficient enough at reducing the viral circulation and the incidence at a highly prevalent farm. This might be due to the relatively low pig density existing in Argentina which suggests that IV may be a good tool to be used in our situation.

Field cases have challenging situations that put pressure on vaccine efficacy, such as increased infection pressure when positive animals are not eliminated, interference from maternal antibodies, and the introduction of positive animals (12). However, the study at the aforementioned farm was very much controlled with the farmer deeply involved, thus allowing for optimal results.

As stated by van Oirschot et al. (1), an intensive vaccination program with a marker vaccine is needed to control AD but must be supported by additional measures, such as replacement with non-infected gilts and culling of infected pigs, making eradication feasible. Based on the current results, we can observe that a good method to eradicate the AD virus is not only the MLV but also the IV method. Vaccination and additional management measures implemented quickly and together allowed for the eradication of AD in the farm under study, and this situation has been sustained over time.

## Conclusion

The applied control strategy based on the vaccination and culling of seropositive animals with certain improvements in biosecurity was suitable for diminishing the incidence and the prevalence at levels compatible with virus eradication.

## Data Availability Statement

The datasets generated for this study are available on request to the corresponding author.

## Ethics Statement

Ethical review and approval was not required for the animal study because the animals in the farm under study were vaccinated and blood samples were taken, with the approval of the farmer.

## Author Contributions

MA and SD conceived and designed the study. FB and RS collected the blood samples. FB performed the ELISA serologic diagnosis. MA wrote the article with support from SD. All authors approved the final version of the manuscript.

## Funding

This study was supported by the INTA Project in Animal Health PNSA 1115057 and Fundación ArgenINTA.

## Conflict of Interest

The authors declare that the research was conducted in the absence of any commercial or financial relationships that could be construed as a potential conflict of interest.

## Publisher's Note

All claims expressed in this article are solely those of the authors and do not necessarily represent those of their affiliated organizations, or those of the publisher, the editors and the reviewers. Any product that may be evaluated in this article, or claim that may be made by its manufacturer, is not guaranteed or endorsed by the publisher.
